# Health of undocumented migrants in primary care in Switzerland

**DOI:** 10.1371/journal.pone.0201313

**Published:** 2018-07-27

**Authors:** Yves Jackson, Adeline Paignon, Hans Wolff, Noelia Delicado

**Affiliations:** 1 Division of Primary Care Medicine, Geneva University Hospitals, Geneva, Switzerland; 2 Global Health Institute, University of Geneva, Geneva, Switzerland; 3 HES-SO University of Applied Sciences and Arts Western Switzerland, School of Health Sciences, Geneva, Switzerland; 4 Division of Prison Health, Geneva University Hospitals and Faculty of Medicine, University of Geneva, Geneva, Switzerland; University of Zurich, SWITZERLAND

## Abstract

**Background:**

Undocumented migrants endure adverse living conditions while facing barriers to access healthcare. Evidence is lacking regarding their healthcare needs, notably in regards to chronic diseases. Our goal was to investigate health conditions in undocumented migrants attended in primary care setting.

**Methods:**

This study was conducted at the primary care outpatient clinic, Geneva University Hospitals, Switzerland. We retrospectively recorded and coded all medical conditions of a random sample of 731 undocumented migrants using the International Classification of Primary Care, 2nd version (ICPC-2). We dichotomized conditions as chronic or non-chronic and considered multimorbidity in the presence of three or more chronic conditions.

**Results:**

Participants originated from 72 countries and were mainly female (65.5%) with a mean age of 42.4 (standard deviation [SD]: 11.4) years. They presented a mean of 2.9 (SD: 2.1; range: 1–17) health conditions. In multivariable analysis, the number of conditions was associated with female gender (p = 0.011) and older age (p <0.001), but not with origin (p = 0.373). The body systems most frequently affected were endocrine, metabolic and nutritional (n = 386; 18.4%), musculoskeletal (n = 308, 14.7%) and digestive (n = 266, 12.8%). Hypertension (17.9%; 95% CI: 15.2%, 29.9%), obesity or overweight (16%; 95% CI: 13.4%, 18.9%) and gastric problems (14.1%; 95% CI: 11.6%, 16.8%) were most prevalent. Overall, 71.8% (95% CI: 68.5%, 75%) participants had at least one chronic condition while 20% (95% CI: 17.2%, 23.1%) had three or more. In multivariable analysis, age (p <0.001) was the only predictor of presenting at least one or three or more chronic conditions.

**Conclusions:**

Undocumented migrants present multiple health problems in primary care settings and bear an important burden of chronic diseases. The extent of multimorbidity highlights the need to provide and facilitate the access to comprehensive and long-term primary healthcare services.

## Introduction

Migration caused by political instability, socioeconomic disparities and environmental events has become a global issue entailing major health challenges, notably access to health services in destination countries. In recent years, Europe has received high numbers of vulnerable migrants due to several geopolitical crises [1]. In Western Europe, migrants granted refugee status or temporary protection are usually entitled to receive basic social rights, including access to the public healthcare system [2]. Undocumented migrants are defined as persons living in a country without a valid residency permit. In 2008, it was estimated that there were 1.9–3.8 million undocumented migrants in the 27 European Union countries [3]. They commonly lack access to basic social services and policies regulating their access to the healthcare system differ between and within countries [4, 5]. Access is frequently limited to emergency care and thus many health services only respond to acute health problems [5]. In addition, access to comprehensive secondary and tertiary care and to costly essential medicines such as antiretroviral drugs is usually severely restricted [6]. Out-of-pocket payment, administrative barriers and fear of denunciation are key factors that impede undocumented migrants to adequately engage with health services. These barriers are associated with potentially severe health consequences for migrants and result in insufficient medical attention to chronic diseases [5–7].

Switzerland hosts 50,000 to 100,000 undocumented migrants, which approximately accounts for 0.6–1.2% of the resident population [8]. They cluster in the main urban areas and are mainly women of childbearing age originating from Latin America, non-European Union European countries, Asia or North/West Africa. They work in the low-wage domestic, hospitality or agriculture industries and endure precarious living conditions [8]. Federal health policies allow them to purchase private health insurance, which is mandatory to access the healthcare system. However, regional disparities in policy implementation and the high cost of insurance premiums (approximately Euro 6000 per year) explain the low proportion of migrants with health insurance and their difficulties to access medical care [9].

Two of the 27 Swiss cantons (Geneva and Vaud) have implemented primary care services within the public healthcare system for this population [9]. Other cantons either delegate this task to nongovernmental organizations (NGO) or offer no service at all, apart from access to the emergency department of public hospitals. Consequently, undocumented migrants receive less preventive healthcare than other groups of the population and management strategies for those with chronic disease are lacking in regions without dedicated primary care programs [10].

Research on vulnerable migrant health needs in Europe has mainly focused on asylum seekers and refugees, while undocumented migrants have received less attention. Most studies were limited to care access and the epidemiology of infectious diseases [5, 11, 12]. Compared to the Swiss general population, previous studies showed that undocumented migrants presented later to antenatal care and had a higher risk of early termination of pregnancy, had a higher risk of sexually transmitted infections in the context of risky behavior, and were more likely to suffer from tuberculosis, Chagas disease and other parasitic infections, but had good coverage against vaccine-preventable infections [13–19]. Most undocumented migrants in Western Europe originate from countries undergoing epidemiological transition characterized by a rapid increase in the burden of non-communicable diseases and their associated risk factors [20, 21]. Therefore, it can be hypothesized that undocumented migrants from these regions present similar health problems. Improved knowledge of the burden of chronic diseases and the extent of comorbidity may help to inform policy-makers and clinicians about optimally designing and delivering healthcare services to this group. We aimed to describe the health conditions found in undocumented migrants receiving care at the Geneva University Hospital.

## Material and methods

### Ethics approval

The study was approved by the ethical research board of the Canton of Geneva (protocol 15–236), which waived the requirement for informed consent given the retrospective design of the study and the full anonymization of the dataset.

### Design

This single center retrospective cross-sectional study used health information extracted from electronic medical records (EMR) of undocumented migrant patients who received care at the Division of Primary Care Medicine of Geneva University Hospitals (HUG) (Geneva, Switzerland) from January 1 to December 31 2014.

### Setting

The Canton of Geneva in Western Switzerland had a population of 490,578 in 2015. An estimated 10,000 to 15,000 undocumented migrants live in the Canton, which represents the highest proportion of a canton population at the Swiss national level [8]. Only 16% have the mandatory private health insurance that allows access to the healthcare system [22]. HUG is the only public healthcare provider in the Canton and offers both comprehensive primary and specialized care to the cantonal population. The community care mobile clinic (CAMSCO) within the Division of Primary Care Medicine is the port of entry for medical services at HUG for vulnerable groups in Geneva, such as those of a low socioeconomic condition, the homeless and undocumented migrants. Except for emergency situations, undocumented migrant patients follow a stepwise itinerary within HUG. CAMSCO nurses conduct a first clinical evaluation, manage benign and frequent complaints according to specific guidelines and provide preventive and screening interventions. In the presence of more complex health needs, they refer patients to the primary care medicine, gynecology or psychiatry outpatient clinics. General practitioners (GP) are in charge of coordinating the subsequent investigations and follow-up. This structured itinerary aims at delivering preventive, curative and rehabilitation care of a standard quality, fostering continuity and cost-effectiveness. In the case of urgent medical conditions, patients are referred to the emergency department and GPs are in charge of the post-emergency follow-up.

### Participants

Eligible participants were identified by searching the HUG administrative database using the following inclusion criteria: a) absence of residency permit in Switzerland; b) age 16 years or older; c) absence of health insurance; and d) having had at least one medical consultation with a GP at the Division of Primary Care Medicine outpatient clinic during the study period.

### Variables

We systematically searched the medical consultations of eligible patients in their EMR to extract the following variables: gender; age; country of origin; and active medical conditions. Conditions included all diagnoses and complaints. Complaints are health problems considered as clinically meaningful by the GP in charge of the patient, but not yet attributed a specific diagnosis. Active conditions are those requiring clinical attention at the time of the medical consultation. GP tag each condition as active or inactive during each consultation (for example, the pneumonia code is active during diagnostic and follow-up consultations and becomes inactive after the consultation confirming clinical resolution). In order to assess the type of care needed, we categorized codes as short-term versus chronic conditions. Chronic conditions were those usually requiring medical attention for 6 months or more or characterized by frequent relapses over a period of years. The decision on the ICPC-2 code categorization was made by consensus between investigators.

### Data source

Each patient is identified by a unique personal number is linked to his/her EMR, which includes all (active and inactive) health problems presented by the patient over time. In the case of multiple contacts with GPs during the study period, we selected the last visit entailing a modification of the list of health problems occurring during the 2014 calendar year. We excluded consultations performed in emergency care settings and only considered GP consultations at the outpatient clinic.

### Measurements

Countries of origin were grouped according to the World Health Organization six regions [23]. All health conditions were coded using the French version of ICPC-2 [24]. This coding is particularly useful in primary care where complaints do not always lead to diagnosis as it classifies problems by body systems and by types. ICPC-2 codes were discussed by investigators to clarify uncertainties about terminology and to ensure consistency and accuracy in coding. A subset of EMR was double-coded for quality control and discrepancies clarified until an agreement was reached.

As the current literature provides no consensus on how to assess multimorbidity in the primary care setting, we used three distinct methods to provide a comprehensive picture of our study population: 1) we counted the number of chronic conditions for each participant; 2) we replicated the methodology used by Pfortmueller et al. among asylum seekers consulting an emergency department of a similar university public hospital in Switzerland [25]. Inspired by the Charlson comorbidity index, it calculates an index score based on 18 severe chronic conditions. Each condition is worth one point; 3) we calculated a score derived from a list of chronic conditions highly relevant to assess multimorbidity in family medicine as recently published by N’Goran et al. [26]. Each of the 75 items was given one point. We considered multimorbidity as clinically significant in the presence of a count of chronic conditions equal or above 3.

### Sample size

We calculated a representative sample size of the population of undocumented patients attended at the Division of Primary Care Medicine (HUG) using a precision of 3% with a confidence level of 95% and included a 3% margin to account for the risk of incomplete or missing information in the medical files. Considering a source population of 2114 persons, we randomly included 731 participants.

### Statistical methods

Descriptive statistics were computed for socio-demographic and health-related characteristics. Age was categorized using quartiles (18–33; 34–41; 42–50; 51 and more years). Continuous variables are presented with the mean, standard deviation (SD) or 95% confidence intervals (95% CI) and compared using Student’s t-test or as median and interquartile range (IQR) and compared with the Mann-Whitney test in the case of non-normal distribution. Categorical variables are presented as percentages and compared using the Chi-square test. We present p-values for trend within age group and region of origin in univariate analysis. Associations between the main outcomes and predictors were analyzed using logistic regressions for binary variables and Poisson regression for non-normally distributed count variables. In these models, gender, age, and origin were used as predictors. We used Africa WHO Region, age quartile 1 and female gender as reference groups. For the count of chronic disease, the Charlson and the N'Goran index, we defined a clinically relevant threshold score of 3 as baselines in the regression analysis models. Statistical significance was set at 5%. All data analyses were performed using SPSS Statistics version 22.0 (IBM, Armonk, NY).

## Results and discussion

### Socio-demographic characteristics

Women accounted for 479 (65.5%) of 731 participants (Table 1). Patients mainly originated from the Americas (n = 398; 54.4%), Europe (n = 114; 15.6%) and the Western Pacific (n = 94: 12.8%). All participants from the Americas originated from countries in South and Central America and to a lesser extent from the Caribbean. Among the 72 countries of origin, Bolivia (n = 132; 18.0%), Brazil (n = 120; 16.4%), Philippines (n = 47; 6.4%), Mongolia (n = 35; 4.7%) and Romania (n = 31; 4.2%) were the most represented. There was a majority of women among participants from the Americas (82. 7%) and Western Pacific (81.9%), while men were more numerous among participants from the other regions. The overall mean age was 42.4 years (SD: 11.4 years) and women were significantly older than men (44.3 versus 38.8 years; p <0.001), but there was no significant age difference between regions of origin (p = 0.51).

**Table 1:** Number of conditions among undocumented migrants (n = 731).

	Participants	Conditions			P-value for trend	Multivariable Poisson regression*	P-value*
	N (%)	Mean (SD)	Range	Median (IQR)		Regression coefficient (95% CI)	
All	731 (100)	2.9 (2.1)	1–17	2 (3)			
Gender					<0.001		0.011
female	479 (65.5)	3.0 (2.2)	1–17	2 (3)		1	
male	252 (34.5)	2.5 (1.7)	1–12	2 (2)		-0.14 (-0.02, -0.25)	
Age					<0.001		<0.001
18–34	172 (23.5)	2.3 (1.7)	1–12	2 (2)		1	
35–42	189 (25.8)	2.5 (1.7)	1–9	2 (2)		0.64 (-0.07, 0.20)	
43–51	183 (25)	3.1 (2.1)	1–11	3 (2)		0.24 (0.12, 0.38)	
53–78	187 (25.6)	3.5 (2.6)	1–17	3 (3)		0.37 (0.24, 0.50)	
Origin					0.512		0.373
Africa	73 (10)	2.6 (1.7)	1–8	2 (2)		1	
Americas	398 (54.4)	3.0 (2.3)	1–17	2 (3)		0.00 (-0.16, 0.16)	
Europe	114 (15.6)	2.6 (1.7)	1–9	2 (3)		-0.01(-0.19, 0.16)	
Eastern Mediterranean	43 (5.9)	2.8 (2.3)	1–9	3 (2)		0.12 (-0.11, 0.34)	
Southeast Asia	9 (1.2)	3.3 (1.7)	1–12	2 (4)		0.27 (-0.11, 0.65)	
Western Pacific	94 (12.8)	2.8 (1.8)	1–8	2 (3)		-0.08 (-0.27, 0.11)	

* Factors included in the multivariable model: gender, age, origin

### Health conditions

Participants presented between one and 17 conditions with a mean count of 2.9 (SD: 2.1) and a median of 2 (IQR: 3) (Table 1). In univariate analysis, the number of conditions was associated with gender (p <0.001) and age (p <0.001) but not with origin (p = 0.512). In multivariate Poisson analysis, older age (p <0.001) and female gender (p = 0.01) remained significant predictors.

### Body systems

**Fig 1:**
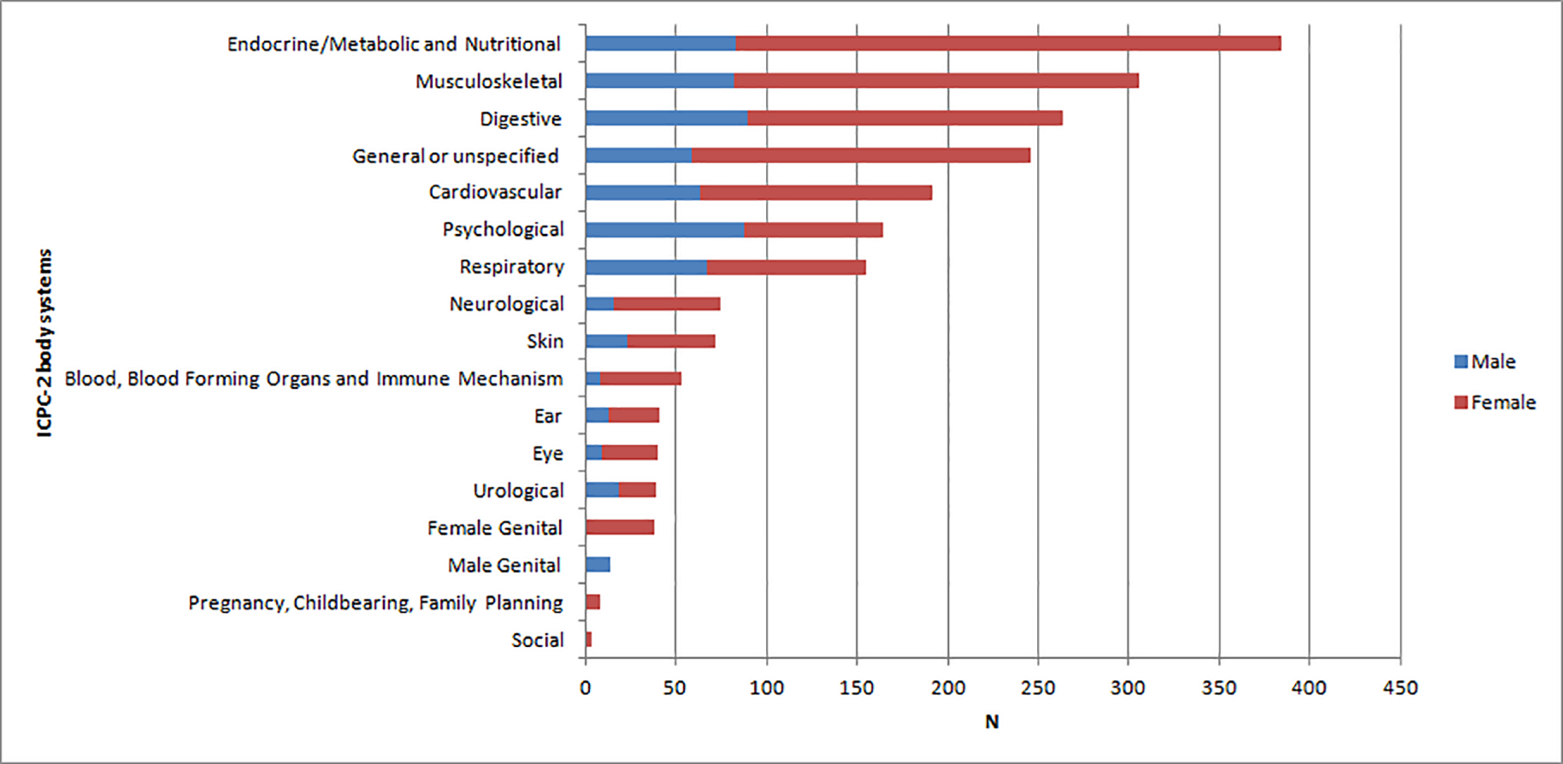
Frequency of conditions categorized by ICPC-2 body system.

ICPC-2 codes predominantly pertained to the endocrine, metabolic and nutritional (n = 384; 18.4%), musculoskeletal (n = 306; 14.7%) and digestive (n = 264; 12.8%) systems. Men predominantly suffered digestive, psychological, endocrine and musculoskeletal problems whereas women mainly presented endocrine, musculoskeletal, general/unspecific and digestive problems (Fig 1).

### Health problems

Hypertension, obesity or overweight, gastric disorders and hypercholesterolemia were the most prevalent health problems among the participants (Table 2). Prevalence the main cardio-vascular risk factors included hypertension (17.9%; 95% CI: 15.2%, 29.9%), hypercholesterolemia (11.8%; 95% CI: 9.5%, 14.3%), diabetes (6.4%; 95% CI: 4.8%, 8.5%) and for tobacco use (5.3%; 95% CI: 3.8%, 7.2%). The prevalence of infectious and psychiatric conditions was low.

**Table 2:** Prevalence and characteristics of the most frequent health problems encountered in male and female undocumented migrants.

Condition	All participants (n = 731)		Female (n = 479)		Male (n = 252)		Chronic conditions
	N	% (95% CI)	N	% (95% CI)	N	% (95% CI)	
Hypertension	131	17.9 (15.2, 29.9)	92	19.2 (15.8, 23.0)	37	14.7 (10.6, 19.7)	yes
Obesity or overweight	117	16.0 (13.4, 18.9)	94	19.6 (16.2, 23.5)	23	9.1 (5.9, 13.4)	yes
Dyspepsia or other gastric problems	103	14.1 (11.6, 16.8)	80	16.7 (13.5, 20.3)	23	9.1 (5.9, 13.4)	yes
Hypercholesterolemia	86	11.8 (9.5, 14.3)	67	14.0 (11.0, 17.4)	19	7.5 (4.6, 11.5)	yes
Vitamin/nutritional or iron deficiency	73	10 (7.9, 12.4)	63	13.2 (10.3, 16.5)	10	4.0 (1.9, 7.2)	yes
Back syndrome with or without radiating pain	58	7.9 (6.1, 10.1)	40	8.4 (6.0, 11.2)	18	7.1 (4.3, 11.1)	no
Constipation or diarrhea	50	6.8 (5.1, 8.9)	30	6.3 (4.4, 8.8)	20	7.9 (4.9, 12.0)	no
Upper respiratory tract infection or other cough	48	6.6 (4.9, 8.6)	27	5.6 (3.7, 8.1)	21	8.3 (5.2, 12.5)	no
Diabetes	47	6.4 (4.8, 8.5)	33	6.9 (4.8, 9.5)	14	5.6 (3.1, 9.1)	yes
Hyperthyroidism or hypothyroidism	44	6.0 (4.4, 8.0)	38	7.9 (5.7, 10.7)	6	2.4 (0.9, 5.1)	yes
Knee symptom/complaint	43	5.9 (4.3, 7.8)	33	6.9 (4.8, 9.5)	10	4.0 (1.9, 7.2)	yes
Anemia	42	5.7 (4.2, 7.7)	36	7.5 (5.3, 10.3)	6	2.4 (0.9, 5.1)	yes
Tobacco use	39	5.3 (3.8, 7.2)	14	2.9 (1.6, 4.9)	25	9.9 (6.5, 14.3)	yes
Migraine or other headache	37	5.1 (3.6, 6.9)	35	7.3 (5.1, 10.0)	2	0.8 (0.1, 2.8)	yes
Rhinitis	37	5.1 (3.6, 6.9)	27	5.6 (3.7, 8.1)	20	7.9 (4.9, 12.0)	no
Abdominal pain	34	4.7 (3.2, 6.4)	26	5.4 (3.6, 7.9)	8	3.2 (1.4, 6.2)	no
Asthma	34	4.7 (3.2, 6.4)	21	4.4 (2.7, 6.6)	13	5.2 (2.8, 8.7)	yes
Anxiety disorder	32	4.4 (3.0, 6.1)	25	5.2 (3.4, 7.6)	7	2.8 (1.1, 5.6)	yes
Depressive disorder	31	4.2 (2.9, 6.0)	20	4.2 (2.6, 6.4)	11	4.4 (2.2, 7.7)	yes
Weakness/tiredness general	29	4.0 (2.7, 5.6)	22	4.6 (2.9, 6.9)	7	2.8 (1.1, 5.6)	yes
Neck symptom/complaint	26	3.6 (2.3, 5.2)	22	4.6 (2.9, 6.9)	4	1.6 (0.4, 4.0)	no
Liver disease or viral hepatitis	24	3.3 (2.1, 4.8)	12	2.5 (1.3, 4.3)	12	4.8 (2.5, 8.2)	yes
Shoulder symptom/complaint	20	2.7 (1.7, 4.2)	13	2.7 (1.5, 4.6)	7	2.8 (1.1, 5.6)	no
Ankle, foot, toe symptom/complaint	20	2.7 (1.7, 4.2)	17	3.5 (2.1, 5.6)	3	1.2 (0.2, 3.4)	no
Vertiginous syndrome	19	2.6 (1.6, 4.0)	14	2.9 (1.6, 4.9)	5	2.0 (0.6, 4.6)	no
American trypanosomiasis (Chagas disease)	17	2.3 (1.4, 3.7)	15	3.1 (1.8, 5.1)	2	0.8 (0.1, 2.8)	yes
Cystitis/other urinary infection	17	2.3 (1.4, 3.7)	11	2.3 (1.2, 4.3)	8	3.2 (1.4, 6.2)	no
Alcohol abuse	16	2.2 (1.3, 3.5)	3	0.6 (0.1, 1.8)	13	5.2 (2.8, 8.7)	yes
Sleep disturbance	16	2.2 (1.3, 3.5)	10	2.1 (1, 3.8)	6	2.4 (0.9, 5.1)	yes

### Chronic conditions

Overall, 1087/2094 (51.9%) conditions were chronic. In total, 71.8% (95% CI: 68.5%, 75.0%) participants had at least one chronic condition. This proportion was significantly higher in women (74.4%, 95% CI: 70.7%, 78.4%) than in men (66.3%, 95% CI: 60.2%, 72.0%) (p = 0.019) but age was the only significant predictor (p <0.001) in the multivariable regression model.

Participants presented an average of 1.5 (SD: 1.5; range: 0–7) and a median of 1 (IQR: 2) chronic conditions. Multivariable Poisson regression showed that age (p <0.001) was the only factor significantly associated with the number of chronic conditions. Patients in age quartile 2 tended to have a higher number of chronic conditions than those in quartile 1 (+0.2; 95% CI: -0.0, -0.4; p = 0.051) whereas patients in quartile 3 (+0.52; 95% CI: 0.33, 0.72; p <0.001) and in quartile 4 (+0.77; 95% CI: 0.59, 0.96; p <0.001) had a significantly higher number of chronic conditions.

### Multimorbidity

The prevalence of participants with three or more chronic conditions was 20% (95% CI: 17.2%, 23.1%) (Table 3). It was significantly higher in women (23.4%; 95% CI: 19.7%, 27.4%) than in men (13.5%; 95% CI: 9.8%, 18.3%) (p = 0.002) and in older age groups (p <0.001) without difference among region of origin (p = 0.247). In the multivariable regression model, age (p <0.001) remained the only significant predictor of multimorbidity.

**Table 3:** Factors associated with multimorbidity in undocumented migrants.

	Prevalence of ≥3 chronic conditions	OR for ≥3 chronic conditions	P-value	Adjusted* OR for ≥3 chronic conditions	P-value*
	% (95% CI)	(95% CI)		(95% CI)	
Gender			0.002		0.220
female	23.4 (19.7–27.4)	1		1	
male	13.5 (9.8–18.3)	0.51 (0.34–0.78)	0.002	0.73 (0.45–1.20)	0.220
Age			<0.001		<0.001
18–34	8.1 (4.7–13.5)	1		1	
35–42	12.2 (8.0–17.9)	1.56 (0.78–3.15)	0.210	1.54 (0.76–3.10)	0.230
43–51	22.4 (16.7–29.3)	3.26 (1.71–6.23)	<0.001	3.12 (1.62–5.99)	0.001
53–78	36.4 (29.6–43.7)	6.45 (3.46–12.02)	<0.001	5.87 (3.12–11.07)	<0.001
Origin			0.256		0.976
Africa	15.1 (8.1–25.8)	1		1	
Americas	22.1 (18.2–26.6)	1.60 (0.81–3.17)	0.178	1.08 (0.52–2.28)	0.831
Europe	14.9 (9.2–23.1)	0.99 (0.43–2.25)	0.977	0.89 (0.38–2.09)	0.793
Eastern Mediterranean	14.0 (5.8–28.6	0.91 (0.31–2.68)	0.870	1.18 (0.39–3.58)	0.770
Southeast Asia	11.1 (2.0–43.5)	0.70 (0.08–6.20)	0.752	0.81 (0.009–7.46)	0.849
Western Pacific	24.5 (16.9–34.6)	1.83 (0.82–4.04)	0.138	1.22 (0.52–2.88)	0.642

* Factors included in the multivariable model: gender, age, origin.

The mean Charlson index score was 0.7 (SD: 0.9; range: 0–5).Women had a non- different mean score than men (0.8 versus 0.7; p = 0.542) and origin was not associated with the score (p = 0.921) in univariate analysis. In multivariate model, age (p <0.001) was the only factor significantly associated with the mean score.

Overall, 5.3% (95% CI: 3.9%, 7.3%) participants had a Charlson score ≥3. Men (6.4%, 95%CI: 3.8%, 10.0%) had a non significant higher prevalence than women (4.8%; 95% CI: 3.1%, 7.2%) (p = 0.390). Age was significantly associated with a score equal or above three (p <0.001) unlike origin (p = 0.291) in univariate analysis and remained significant in the multivariate model (p <0.001).

The mean N’Goran score was 1 (SD: 1.1; range: 0–7) without difference between women and men (1.1 versus 1.0; p = 0.211) and regions of origin (p = 0.682) in univariate analysis. In the multivariable analysis, this score was only significantly associated with age (p <0.001). The overall prevalence of N’Goran score ≥3 was 9.4% (95% CI: 7.3%, 11.6%), 8.3% (95% CI: 5.5%, 12.4%) in men and 10% (95% CI: 7.6%, 13.0%) in women (p = 0.453). Age (p <0.001) was the only significant predictor in the multivariable regression model.

## Discussion

This study explores the health profile and healthcare needs among undocumented migrants in a primary care setting in Europe and is the largest conducted so far in this population to the best of our knowledge. The middle-aged, mainly female and non-European patients presented cumulative health conditions of diverse types, with a majority requiring long-term medical attention. We found a high prevalence of cardiovascular risk factors and between one in five and one in twenty participants presented with multimorbidity depending on the methods of assessment. Whereas gender and origin did not influence healthcare needs, older age was associated with a higher burden of disease.

Previous studies showed that undocumented migrants had a worse perceived health status than the general population [27, 28]. In our study, we found that participants suffered three concomitant health problems on average with women and older patients presenting more conditions. This is much higher than previously reported in other vulnerable migrant groups in Switzerland. Pfortmueller at al. found a mean disease count of 0.58 among 3170 asylum seekers consulting at the emergency department of a university hospital [25]. Bischoff et al. reported a median number of zero (IQR 3) diagnoses among asylum seekers in a health maintenance organization [29]. A similarly designed study among prisoners (92.8% migrants) using the ICPC-2 classification found a mean count of 2.4 (SD: 1.8) conditions [30]. Our findings have to be appraised in light of the fact that undocumented migrants usually consult less and later in the course of health problems than the general population and, notably, asylum seekers and refugees who have easier access to the Swiss healthcare system [13]. The high number of health conditions in this relatively young population lends support to the fact that adverse living conditions, including precarious working conditions, and the absence of legal entitlement to social rights may negatively impact on health and calls for reinforced and targeted preventive and screening health strategies for this group [27]. One question that remains to be elucidated relates to the impact of migrants’ regularization on general health and well-being.

Endocrine, metabolic and nutritional along with musculoskeletal and digestive conditions predominated in our sample, which confirms previous findings among undocumented migrants [28]. It is noteworthy that infections represented only a minor part of conditions, which contrasts with findings in other migrant populations, such as among detainees in Switzerland [30]. The low prevalence of mental health conditions was also dissimilar to other migrant groups. Conflicting evidence exists about undocumented migrants’ mental health. While some studies showed they suffered less anxiety and depression than asylum seekers and refugees, others have highlighted the frequent pathogenic impact of social exclusion and material deprivation [27, 31, 32]. One explanation for this finding may relate to the fact that the GPs in charge of the patients were mainly residents who could have overlooked such problems in the context of cumulated conditions. Theunissen et al. showed that even experienced practitioners faced difficulties in accurately recording mental health problems in migrants with multiple somatic conditions [33]. Indeed, the ability to correctly diagnose mental health problems in migrants is frequently hampered by social, cultural and structural factors influencing patient-doctor interactions [34]. A second hypothesis pertains to the frequent lack of health insurance and therefore potential to access to effective therapeutic intervention, which may prevent clinicians from investigating mental health as thoroughly as among other migrants groups. This calls for ensuring adequate training in mental health screening and management for practitioners involved in migrant healthcare and removing barriers to treatment for patients in need of therapeutic interventions.

We assessed multimorbidity using two index scores to compare our results with previously published data in Switzerland and by counting the number of chronic disease. We found a mean Charlson index of 0.7, which contrasted with 0.25 among asylum seekers [25]. The N’Goran index mean score was 1. In a study conducted among older (mean age: 73 years) patients in a primary care setting in Switzerland, the mean score amounted to 5.5 [35]. The prevalence of multimorbidity using the Charlson and N’Goran scores (5.3% and 9.4%, respectively) underestimated the 20% prevalence calculated from the total count of chronic diseases, highlighting the difficulty to correctly report multimorbidity. These figures confirm the growing body of evidence about the burden of chronic diseases among undocumented migrants in Europe. It also points to the methodological difficulties to reliably and consistently assessing it in primary care. A survey conducted among 1218 migrants in 11 countries showed that 32% suffered at least one chronic condition [28]. In a clinical setting similar to Geneva, chronic diseases accounted for 22% of conditions with 6.5% patients having three or more conditions [36]. An indirect confirmation was brought by Fiorini et al. showing that drugs for chronic conditions (hypertension, hypercholesterolemia and diabetes) were the most frequently prescribed in a large cohort of undocumented migrants in Milan (Italy) [37]. Indeed, we found that cardiovascular risk factors, such as hypertension, obesity and hypercholesterolemia, were among the five most frequent conditions in our sample. This finding has significant clinical and public health consequences. Undocumented migrants have a higher risk of hospitalization for preventable complications of chronic diseases [38]. Most migrant groups show higher diabetes-related mortality and undocumented migrants have a two-fold cardiovascular mortality rate compared to the general European population [39, 40]. Screening and active management of cardiovascular risk factors should therefore be a priority in this population.

Moreover, we found that 51.4% conditions required long-term medical attention. In Europe, access to care for undocumented migrants often rely on NGOs or is restricted to the emergency department in public hospitals. Our findings have two main implications. First, they call for planning health services to undocumented migrants in a way that ensures provision of long-term, multidisciplinary and comprehensive care [4, 41]. Second, these services imply substantial financial investment over the long term that may not be sustainable for NGOs. Therefore, integration into the public healthcare sector is warranted in order to ensure equity and sustainability in access to quality comprehensive medical care. Alternatively, restricting access to the public healthcare system to vulnerable migrants entails higher global financial costs as recently shown in Germany [42]. Experiences in Switzerland and The Netherlands show that such a scheme is feasible, equitable and effective in regards to the management of chronic diseases [43, 44].

The main strength of this study is the large random sample of undocumented migrants in a primary care setting, a population notably hard to reach and poorly investigated so far. Moreover, the access to patient information through EMR allowed for comprehensive data extraction. However, there are several limitations: a) the extensive diversity of undocumented migrant populations across different settings in Europe restricts the generalizability of our findings; b) the predominance of women in our sample impacted on the global health needs, although this may differ in regions where men predominate; and c) the retrospective cross-sectional design did not allow for systematically exploring important variables such as health behavior.

## Conclusions

Our findings suggest that undocumented migrants frequently present with cumulative health problems with an important burden of chronic and multimorbid conditions. This highlights the need for enhanced awareness and specific attention from health care professionals and policy-makers to ensure the planning and delivery of comprehensive primary care services.
